# Supplementary material for: Mitochondrial Heteroplasmy and PCR Amplification Bias Lead to Wrong Species Delimitation with High Confidence in the South American and Antarctic Marine Bivalve Aequiyoldia eightsii Species Complex
Source: Genes (Basel). 2023 Apr 18;14(4):935. doi: 10.3390/genes14040935 (PMC10138075; doi:10.3390/genes14040935)

Supplementary Materials - S1

Table S1. Annotation of 18 kb- mitochondrial transcript. Annotation of 18 kbp transcript (TRINITY_DN0_c0_g1_i6) using MITOS (Bernt et al. 2013). Above: The table provides the name of the mitochondrial gene annotated, the start and stop position, the direction of the strand and the length of the gene. Below: Mapping of genes on 18kb transcript (red: protein coding gene, green: rRNA gene, blue: tRNA gene)

Name	Start	Stop	Strand	Length
nad3	1283	1615	+	333
trnG(gga)	1654	1716	+	63
nad2	1777	2604	+	828
cox1	2890	4368	+	1479
nad6	4625	5107	+	483
trnK(aaa)	5155	5222	+	68
trnL1(cta)	5226	5291	+	66
trnE(gaa)	5317	5385	+	69
trnY(tac)	5390	5454	+	65
trnF(ttc)	5463	5528	+	66
trnH(cac)	5555	5623	+	69
rrnS	5653	6587	+	935
rrnL	6571	7935	+	1365
trnM(atg)	7946	8014	+	69
trnP(cca)	8020	8085	+	66
nad5	8126	9769	+	1644
trnM(atg)	9794	9861	+	68
trnS1(aga)	9877	9937	+	61
trnT(aca)	9951	10019	+	69
cox3	10142	10888	+	747
nad1	10990	11871	+	882
atp6	12176	12625	+	450
nad4l	12731	12964	+	234
nad4	13082	14290	+	1209
trnN(aac)	14322	14389	+	68
cob	14445	15527	+	1083
cox2	15646	16293	+	648
trnQ(caa)	16328	16396	+	69
trnS2(tca)	16401	16463	+	63
trnW(tga)	16482	16550	+	69
trnA(gca)	16562	16629	+	68
trnL2(tta)	16842	16907	+	66
trnR(cga)	17045	17114	+	70
trnV(gta)	17139	17206	+	68

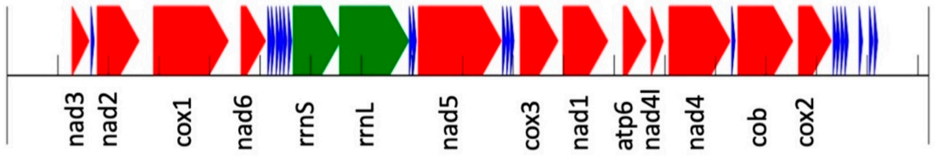

Table S2. COI p-distance. p-distances based on *COI* between the most frequent haplotypes of *Aequiyoldia* found in Punta Arenas, Potter Cove and Hangar Cove.

	h1	h2	h3	h4
h1	*			
h2	0.8	*		
h3	6.1	5.9	*	
h4	7.0	6.6	8.6	*

Figure S1. **Shell length of *Aequiyoldia.*** Box plot of Shell length of *Aequiyoldia eightsii* based on locations (Antarctica and South America) (left) and mitotypes (right). Box plot based on location includes all the animals collected and measured, while the boxplot based on mitotypes includes only those animals analyzed with haplotype-specific primers. The thick line indicates the median, the box represents the first and third quartile, and whiskers the 95 % confidence interval

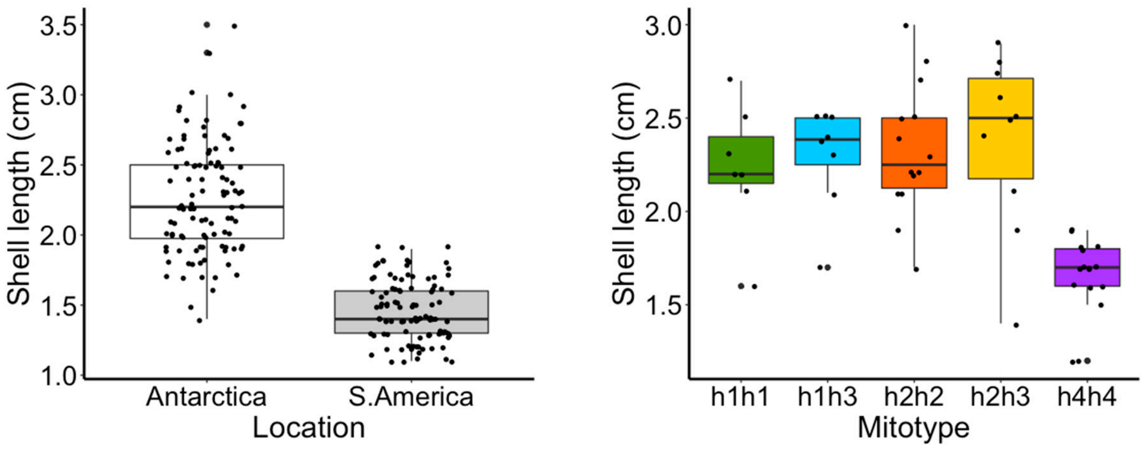

Supplement: Supplementary file 1 [file genes-14-00935-s001.zip › genes-2283944-supplementary.pdf]
